# Experimental Diagnostics of the Emotional State of Individuals Using External Stimuli and a Model of Neurocognitive Brain Activity

**DOI:** 10.3390/diagnostics12010125

**Published:** 2022-01-06

**Authors:** Alexandr Y. Petukhov, Sofia A. Polevaya, Anna V. Polevaya

**Affiliations:** 1 Keldysh Institute of Applied Mathematics RAS, 4 Miusskaya Square,125047 Moscow, Russia; 2 Department of Psychophysiology, Lobachevsky State University of Nizhny Novgorod, Nizhny Novgorod 23 Gagarina Ave., 603105 Nizhny Novgorod, Russia; s453383@mail.ru (S.A.P.); a.dostoevskaya@gmail.com (A.V.P.)

**Keywords:** emotions, experimental modeling, diagnostics, neurocognitive activity of the brain, information images, representations, heart rate telemetry

## Abstract

In this paper, we study ways and methods to diagnose the emotional state of individuals using external audiovisual stimuli and heart telemetry tools. We apply a mathematical model of neurocognitive brain activity developed specifically for this study to interpret the experimental scheme and its results. This experimental technique is based on monitoring and analyzing the dynamics of heart rate variability (HRV), taking into account the particular context and events occurring around the subject of the study. In addition, we provide a brief description of the theory of information images/representations used for the paradigm and interpretation of the experiment. For this study, we viewed the human mind as a one-dimensional potential hole with finite walls of different sizes and an internal potential barrier modeling the border between consciousness and subconsciousness. We also provided the foundations of the mathematical apparatus for this particular view. This experiment allowed us to identify the characteristic markers of influencing external stimuli, which form a foundation for diagnosing the emotional state of an individual.

## 1. Introduction

Heightened stress states and emotional negativization can significantly affect the psychological and physiological state of individuals. This problem becomes exponentially more relevant in today’s networked world. The reasons for these adverse effects can be both external (pandemic, wars, migration crises, etc.) and internal, such as from increased social pressure. The latter occurs because modern individuals become increasingly more integrated into a variety of different information processes, which, in turn, produce a growing number of corresponding external stimuli.

This is why it is important to have the necessary tools for diagnosing the emotional state (and related phenomena, such as emotional maladjustment, increased anxiety, etc.) of the individual. Given the scale and prevalence of the phenomenon, such a diagnosis has to be large-scale and simple to implement without significant loss of accuracy. 

Numerous methods and approaches, for example, the SAM (Self-Assessment Manikin-) questionnaire [1,2], identifies emotions by linking physiological and self-reported indicators. 

There are also several works devoted to the use of IAPS in multimodal neuroimaging, for example–[3].

All of these methods often rely on the use of images or, in some cases, sounds (music, sounds of nature) as a stimulus material [4,5,6,7,8]. In addition, scholars actively use modern neurofeedback technologies that utilize resonance stimulation of the endogenous rhythmic activity of the brain. These methods can induce certain functional states in an individual, for example, to correct disorders of the nervous system [9] or improve an individual’s adaptation to the environment by binaural stimulation [10]. 

The most well-studied neurofeedback mechanisms are the facial muscle contraction and electrical activity of the skin [11]. Several studies have attempted to get the required feedback directly from the brain, but at the moment they have not been able to produce unambiguous results [12]. Izard, C. E. defines emotions as “a complex phenomenon that includes neurophysiological, motor, and expressive components, as well as subjective experience” [13]. The interaction of these components in the intraindividual process forms an emotion–an evolutionary biogenetic phenomenon.

Some researchers [14] consider emotions as a complex psychophysiological phenomenon that can be associated with:

(1) experienced and/or perceived feeling (state)-the phenomenology of emotions;
(2) visceral processes accompanying emotions;
(3) expressive characteristics of emotions (facial expression, intonation, gestures, and postures).

Accordingly, emotions can be used as a particular tool/marker for the development of an appropriate model and scheme for initiating external stimuli and diagnosing characteristic states of an individual, including the emotional states.

Moreover, one of the aspects of human cognitive activity is the fact that individuals do not think in codes (similar to a computer); our mind operates with multiple interacting images/representations. These images have a very specific physical structure at their functional core (namely, electrical and chemical activity in the human brain). However, their description from the point of view of conventional mathematical models is difficult for a number of reasons [15,16]. 

Therefore, to develop an appropriate experimental scheme, we need to propose a model capable of correctly displaying the dynamics of information images/representations during the cognitive activity (expressed in the neural activity of a set of synapses and patterns) and give this model a conceptual and mathematical embodiment. This model should qualitatively explain the induced processes in the context of the experiment and predict/interpret the final result to deliver the diagnosis. This article proposes new methods for describing the activity of information images (which, in turn, model the neurocognitive activity of an individual) based on the mathematical apparatus of quantum physics (potential holes and virtual particles used in physics to describe fundamental interactions [17,18]).

## 2. Experimental Approaches to Diagnosing Emotions

The following basic methods are used for diagnosing emotions:

1. Analysis of brain activity (EEG, brain mapping);
2. Analysis of vegetative and somatic signs of emotional states (electromyography of the face-stimulation of facial muscles, electrical activity of the skin, body temperature, pupil dilation, eye-tracking-analysis of oculomotor movement, blinking on startle, posterior reflex, analysis of heart rate variability).

An important parameter in psychological diagnostics is ambivalence, which allows researchers to keep the subject focused on the experiment without any distractions and, at the same time, obtain reliable data. One of these methods is the analysis of heart rate variability. A heart rate variability study includes tracking the heart rate, the average duration of the RR interval, the number of RR-interval-pairs, and the power of the high-frequency band [2,11,19].

HRV is the time interval from the start of a cycle of one heartbeat to the start of another heartbeat. This interval is constantly changing and can be observed even during rest in a supine position. HRV indices are reliable data for identifying the tone of the ANS (autonomic system, its sympathetic and parasympathetic divisions) and for analyzing the controllability of physiological functions of the body. Scholars use HRV to assess the changes and dynamics of the psycho-emotional state of an individual [20].

Analyzing emotions using heart rate variability has several advantages. It is one of the most convenient indicators–it is based on the performance of the cardiovascular system of the body. Another advantage of this method is that the necessary data can be obtained via a small sensor. This sensor is easy to put on and it does not hinder the movement of a subject. The subject can even forget about a sensor, which will further minimize the impact of the experiment on their natural state, thereby, making the obtained data more accurate [21,22,23]. 

The novelty of the proposed experimental approach lies in the fact that we use methods suitable for ambient (background) monitoring (ambient registration of physiological signals), which do not involve human cognitive resources at the time of registration [24].

To study the effects of auditory and visual analyzers, most researchers use the IAPS (developed by P. Lang and consists of 1182 images) and IADS (developed by M. Bradley and in the second edition consists of 168 sound compositions [25,26,27,28]) databases. 

These photos and sounds have a wide variety of tones and scenarios, and represent numerous contexts. The sounds and photos, therefore, are recognizable by subjects from different countries and can trigger similar emotional reactions. To assess the perception of the stimuli by individuals, the authors of the abovementioned databases used the SAM method (Cook III EW, 1987). The advantage of this technique is that it is simple, can be easily used by all subjects, and can even be applied to children. In the original SAM method, emotions are assessed on three scales: (1) by valence (sign),from extremely negative (1-despair, depression) to extremely positive (9-joy, euphoria), (2) by arousal (somatic)-from relaxed (1) to active (9), and (3) by the ability to control emotion-from complete lack of control (1) to complete control (9). The data were labeled on a large sample based on the assessments of the subjects. 

The effectiveness of the application of stimuli from this standard database has been shown in numerous laboratories around the world [27,28]. The set of photographs and images presented in the database covers a variety of aspects of the human emotional sphere and can trigger complex subjective and autonomic emotional reactions that vary depending on the valence and activating content of the stimulus. 

The key methodological problem of the study of emotions using subjective assessments boils down to the fact that these assessments can be influenced by many external factors, such as gender, cultural affiliation, and other personal characteristics of the individual [29]. 

In addition, the validity of subjective reports is strictly limited in time: the longer the time between the assessment of the state and the emotional event, the less its reliability. In this regard, the study of the emotional sphere of a person should rely on the objective methods for assessing emotions obtained both from the central (EEG) and from the autonomic nervous system (ECG, GSR, respiration). With an integrated approach to the study of emotions, special attention should be paid to the assessment of the behavioral reactions of the subject. 

The cognitive component of the assessment of emotions is based on assessing the stimulus and individual’s state both before and after exposure to a stimulus. These databases proved to be effective for studying HRV and emotional manifestations triggered by stimuli of different valences. However, the specifics of the sample should not be disregarded. Studies show that stimuli of different valence modify the initial state of the individual differently. Therefore, we can assume that the study of heart rate variability can help track the occurrence of emotions and possibly even their valence. 

The Self-Assessment Manikin (SAM) Questionnaire (Cook III EW, 1987) is a model for self-assessment of emotions. It involves a non-verbal assessment of three emotional states: the sign of emotion or valence (valence), the strength of the emotion (arousal), and the impact on self-esteem or dominance (dominance). The technique includes three rows of pictures (one row for each of the emotions), each containing five degrees of manifestation of emotion from “low” to “high”. 

Sometimes subjects find it difficult to describe their emotions. The reasons for that could be semantic (incomplete understanding of meaning) or lexical (does not know how to describe the manifested emotion), etc. Therefore, the preferred way to determine the actual genesis of emotions of individuals in the sample is to use standardized express methods based on pictographic design. This will simplify the correlation of the actual state of the subject and the parameters of the method (scale). 

Let us move on to our model representation of the neurocognitive activity of the brain and the way it interprets an individual’s emotional response to external stimuli.

## 3. The Foundations of the Theory of Information Images/Representations and Its Model

The proposed theory is based on the idea of a universal cognitive unit [15] of information in the human mind-an information representation (or image), the space in which it exists, its topology, and properties. Information representations (hereinafter referred to as IRs) can be defined as displays of objects and events in a given space.

Correspondingly, the theory of information representations (hereinafter referred to as TIR) is a way to describe information interactions of individuals, as well as several cognitive functions of a person.

TIR views the human mind and neurocognitive activity of the brain as a large structured number of interacting images which are constantly affected by external factors.

Images with higher energy (we introduce the notion of energy E to describe the communicative activity of images) are located “above” and closer to the edge of the individual’s information image space, which is why they interact with external elements much more often. Meanwhile, images with lower energy and longer response rate are located closer to the center of the space and relatively rarely interact with external stimuli.

TIR gives an alternative perspective on some characteristic regularities in the human mind, allowing for a correct interpretation and explanation of some of them.

The information image cannot be communicated between individuals without changes. Each IR is unique because each individual has specific individual experiences. 

Besides, an individual is unable to convey the image that exists in their mind, in their IR space to another individual directly. For this purpose, they use various communication apparatuses formed within the social superstructure of communication or the communication field (CF). A communication field (CF) is an information-based community of individual experiences and collective unconsciousness formed as a result of an individual’s presence in society. The communicative apparatus includes speech, visual, tactile, symbolic, and other ways of information transmission.

In previous works, the mathematical model of interaction of information representations was based on diffusion equations (first of all, on the Langevin equation). This approach allowed us to describe several special cases of cognitive processes but also had some limitations related to the specificity of IRs. More details about TIR and its application to real cognitive processes and experimental testing are available in [16].

Additionally, the interaction of individuals is a process of emission and absorption (processing and perception) of IRs. Thus, we can describe any information interaction as a result of virtual processes related to IR.

The classical method for describing a virtual process (a process involving virtual particles) is the Feynman diagram method [17]. However, the direct application of such a device for IRs would hardly be possible. 

**Figure 1:**
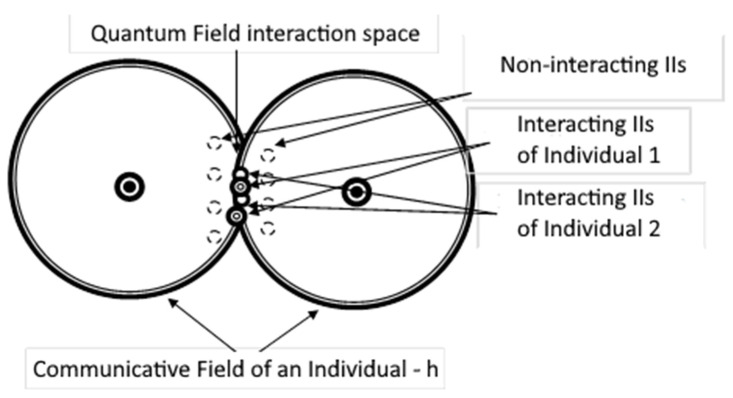
Interaction of communicative fields of two individuals.

Within the model, the interaction of two individuals is represented as the interaction of two systems with the help of communication fields in the information environment (Figure 1). Also, of interest is the impact of a given information environment (e.g., media, Internet resources, social environment, etc.) on an individual.

The interaction function for these two individuals or an individual and the external action should be written accordingly.

Since we are talking about the IR space, it is obvious that this space will have certain coordinates, and the particles simulating the IR movement in this space will also have a certain momentum, energy. At the same time, we should keep in mind the presence of local minimum potential particle energy in this space. Thus, a mind can be represented as a potential well, inside of which information representations make vibrational movements. In physics, we commonly solve the problem of quantization of the field inside the potential well to determine energy levels (by solving the stationary Schrödinger equation [18]). However, in this case, we know about the presence of particles in advance.

**Figure 2:**
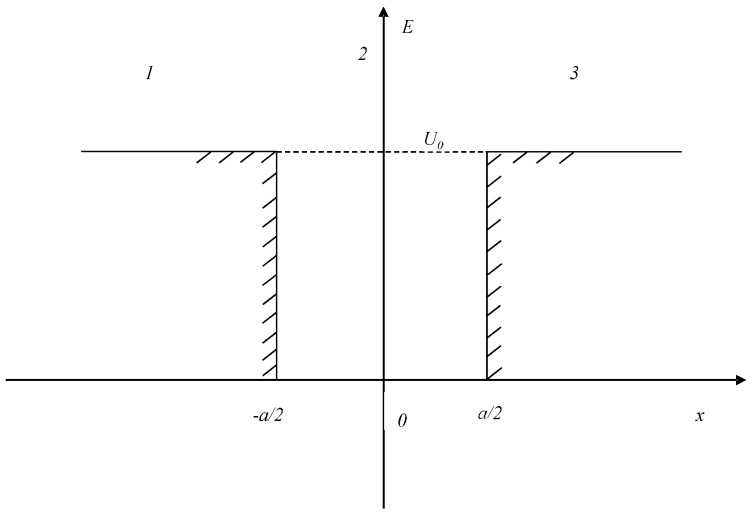
The most basic one-dimensional potential well with finite walls.

The potential well can be represented in the simplest form as–Figure 2.

(1) $$U\left(x\right)=\{\begin{array}{c} 0,-\frac{a}{2}<x<\frac{a}{2},\;\mathrm{region}\;2\\ U_{0},\begin{array}{c} x\geq \frac{a}{2},\;\mathrm{region}\;3\\ x\leq -\frac{a}{2},\;\mathrm{region}\;1 \end{array} \end{array}$$

The value-*U*(*x*) describes the potential energy profile in the well. In this model, the indicator of potential energy reflects the levels of activity of information images. This is directly related to the movement of images. The higher levels correspond to IR, which are now in an activated state due to cognitive activity, while low levels represent images/representations that do exist in individual’s mind, but have not been activated for a long time, which means they remain in the subconscious zone. That is, IR with high energy are determined by the set of thoughts of a person at the moment, and with IRs with low energy are responsible for information processes in the subconscious.

When a particle crosses the potential barrier in the form of a wall, it simulates the information interaction of an individual (i.e., particle disturbance, the transition from one energy level to a higher one).

**Figure 3:**
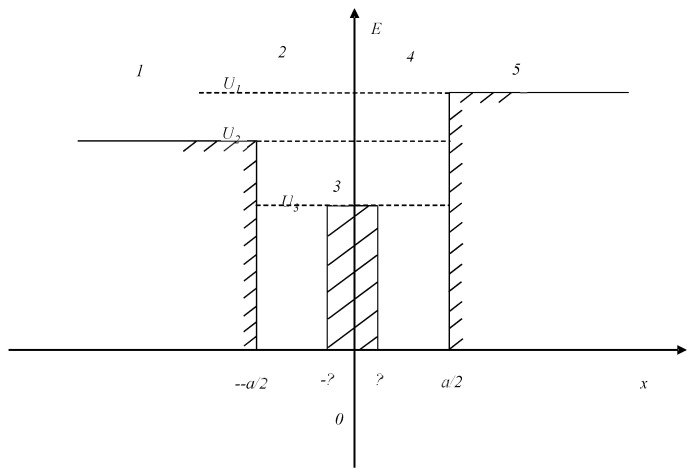
One-dimensional potential well with finite walls. A version with uneven walls and an internal potential barrier.

A more interesting case of a potential well is the well with uneven walls and an additional internal barrier. Such barrier and heterogeneity of walls simulate certain specific properties of the human mind, in particular, the conditional division into consciousness-subconsciousness, complexity of interaction with the external environment, etc. Theoretically, depending on the mental state of the modeled individual (or their individual cognitive function), such barriers may be more complex and repeated multiple times–Figure 3.

(2) $$U\left(x\right)=\{\begin{array}{c} U_{1},\;x\geq \frac{a}{2}\\ 0,\;\varepsilon <x<\frac{a}{2}\\ U_{2},-\varepsilon \leq x<\leq \varepsilon \\ 0,-\frac{a}{2}<x<-\varepsilon \;\;\\ U_{0},\;x\leq -\frac{a}{2} \end{array}$$

Thus, the impact of the external information environment on the mind of an individual can be described as the impact on particles (modeling information images) in a potential well. Let us record the equations for the case in Figure 2.

The Schrödinger equation outside the potential well for the information image (IR) will look as follows:

(3) $$\frac{d^{2}\varphi_{out}\left(x\right)}{dx^{2}}-\frac{2m}{\hbar^{2}}\left(U_{0}-E\right)\varphi_{out}=0\;\;\;\;$$

where,

$\varphi_{out}$ is a wave function outside the potential well.

*E* is the total energy of an IR.

*m* is mass (complexity) of IR.

ℏ is the Planck constant.

By introducing the interaction coefficient *k*_1_,

(4) $$k_{1}=\sqrt{\frac{2m}{\hbar^{2}}\left(U_{0}-E\right)}$$

We obtain,

(5) $$\frac{d^{2}\varphi_{out}\left(x\right)}{dx^{2}}-k_{1}{}^{2}\varphi_{out}=0\;\;\;\;\;\;$$

The external impact will be recorded by disturbance *V*(*x*), which is added to the general form of the Schrödinger equation:

(6) $$\frac{d^{2}\varphi \left(x\right)}{dx^{2}}-\frac{2m}{\hbar^{2}}\left(U\left(x\right)+V\left(x\right)-E\right)\varphi =0\;\;\;$$

Disturbance can be set in different ways, depending on its type, for example, by normal distribution or other methods.

More details about the model and TIR are available in [16,18].

In this interpretation, individual’s response to the informational impact will be presented in the form of activation (i.e., moving the image/images) from a relatively low level to a higher one. From the point of view of the TIR, images used unconsciously have significantly greater inertia, which leads to a relatively large amount of time for their use in conscious communication. At the same time, the human brain, as a rule, tends to choose the closest images for interaction.

**Figure 4:**
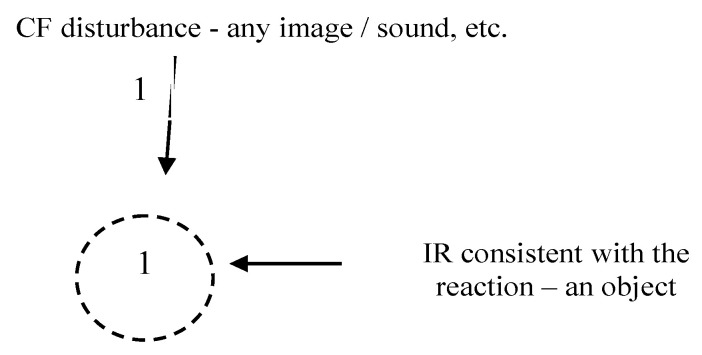
Stimulus.

The impact in terms of TIR is as follows (Figure 4):

The figure shows an external stimulus (one or many).

As a result of the excitation of the IR, it (IR) moves to a higher energy level. The time of its activation corresponds to the time of decision-making by the individual and/or the psychophysiological reaction of the individual, changes in their functional state, including the emotional reaction corresponding to their state (including stress). Therefore, during the experiment, it is necessary to create a stable and statistically reliable relationship between the psychophysiological response to a provoking stimulus and the presence/absence of changes in the emotional state. 

Now, it is necessary to discuss the features of our experimental approach to identifying such markers.

## 4. Experimental Methods

**Figure 5:**
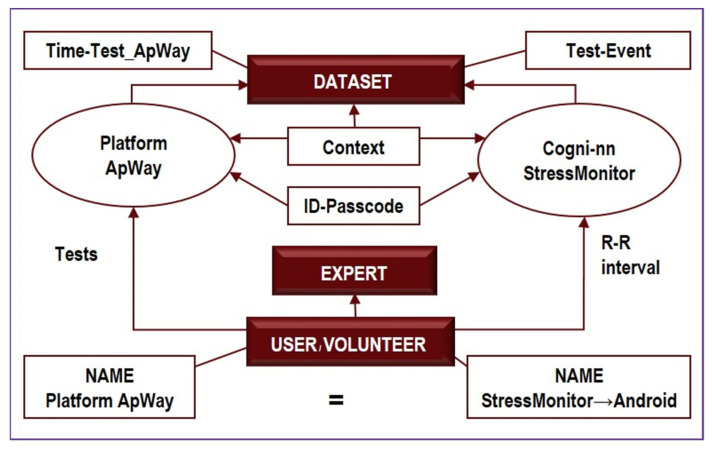
The architecture of the event-driven telemetry.

The method of event-related telemetry of the heart rate [24]. The technology provides monitoring and analysis of the dynamics of heart rate variability (HRV) taking into account the event context. The sequence of time intervals between heartbeats (rhythmogram) is recorded with chest plastic electrodes. Primary signal processing and data transmission to a smartphone is performed by the Zephyr HxM ™ Smart Heart Rate Monitor (HxM, Zephyr Technology, New Zealand) via Bluetooth. The specialized Android (4.4 and above) application “Stress monitor” performs the real-time monitoring and transfers data to the cloud server. Rhythmographic visualization, spectral analysis, and detection of stress episodes are implemented in the specialized online-service “Stress Monitor” (cogninn.ru) [5]. The architecture of the event-based telemetry technology is shown in Figure 5.

Event-related telemetry uses specialized hardware and software for detecting early biomarkers of extreme conditions in real-time. This complex neither limits the mobility of a test subject, nor attracts their attention to the process. To collect telemetry data and detect stress, the technology uses the dedicated StressMonitor application based on cogni-nn.ru. The controlled activation of primary cognitive functions in the contexts of sensorimotor activity is done by a Web-platform at platform.apway.ru. 

This technique enables continuous long-term collection, transmission, accumulation, and preprocessing of time-synchronized cardiac rhythmographic records, spacial data on the trajectory of a person’s movement in a room and open space, video, and audio surveillance data, and the results of psychophysiological tests. 

In our case, this technique helps us determine the functional states of the subjects. It analyzes the way subjects function and adapt to changing environmental conditions and determines their functional level. This analysis is based on the following parameters:

(1) Average heart rate (HR);
(2) Average RR-intervals;
(3) Average power of the low-frequency band of the total HRV spectrum (LF);
(4) Average power of the high-frequency band of the total HRV spectrum (HF);
(5) Average ratio of slow and fast bands of the general HRV spectrum (a measure of sympathovagal balance) (LF / HF);
(6) Average power of the HRV spectrum (TP);
(7) Stress Index (SI);
(8) Functional reserve (FR);
(9) Stress Index (SN).

Our study focuses on their combination in response to audiovisual stimulus material. 

It is important to determine those individual physiological markers recorded during day-to-day activity which could serve as indicators of emotional maladjustment, i.e., emotional stress and emotional exhaustion. Information about these markers can give individuals timely feedback and demonstrate the level of their current mental stress. Subsequently, the individual can temporarily reduce this stress by switching to physical or other types of activity. In this case, we used the method of assessing the level of emotional maladjustment for diagnostics of individual states [30].

## 5. Experiment and Sampling

The samples used in this study are presented in the Appendix B.

**Figure 6:**
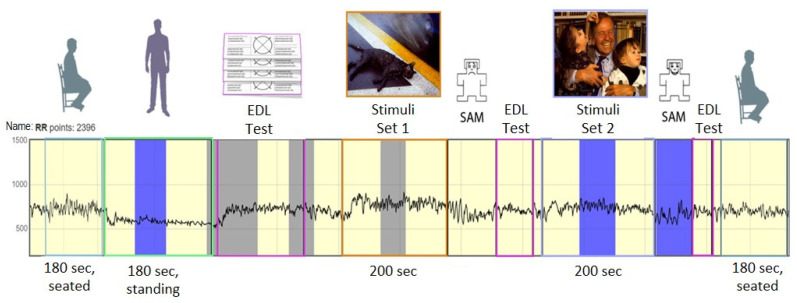
Basic experiment design.

The study was divided into two stages (Figure 6): the preparatory, where we standardized stimulus sets, and the experimental, where we studied connections between the autonomic and cognitive assessment of emotions.

### 5.1. Preparatory Stage:

The stimulus material for the study was selected from the IADS and IAPS databases. 

1. The experimental model for the registration of cognitive manifestations (assessment) of affective sounds included 30 sets selected according to their compliance with the criteria of the SAM (Appendix A) Valence scale (Pleasure):

- 10 positive (with the highest average values-from 2.33 to 2.9),
- 10 neutral (with the values closest to zero-from −0.14 to 0.15).
- 10 negative (with the smallest values-from −3.43 to −2.96)

The average indicators according to the SAM method were taken based on the research of R.A. Stevenson conducted on American subjects [28].

The study involved 83 people aged 17 to 24, nine of whom were men, and 74 women. All participants had no problems associated with cardiovascular, psychiatric, respiratory diseases, and they did not take any medications. All subjects were informed and signed informed consent to participate in the study.

The study was conducted in a room isolated from excessive noise and with a comfortable workplace. The study conditions met the basic requirements for psychological testing.

At the very beginning, the subjects were asked to familiarize themselves with the informed consent to participate in the study. After that, the subjects were asked to randomly listen to 30 recordings and give each a score on the SAM scales.

2. Development of an experimental model for cognitive manifestations (assessment) of affective images included 84 photographs from IAPS. The photos were selected according to their compliance with the criteria of the Pleasure scale of the SAM method.

- 28 positive (with the highest mean values ranging from 2 to 4).
- 28 neutral (with values closest to zero-from −1 to 1).
- 28 negative (with the smallest-from −4 to −2)

The average SAM values were taken based on a study by P. Lang conducted on American subjects [26,27]. 

The study involved 21 people aged 20 to 24, 12 women and nine men. No participants had any cardiovascular, psychiatric or respiratory diseases, and they did not take any medications. All subjects were informed and signed informed consent to participate in the study.

The study was conducted in a room isolated from excessive noise and with a comfortable workplace. The study conditions met the basic requirements for psychological testing.

At the very beginning, the subjects were asked to familiarize themselves with the informed consent for participation in the study. Subsequently, the subjects assessed 28 positive, 28 neutral, and 28 negative images using the SAM rating scales. 

Before each photograph was displayed for the assessment, there was a break of 5 s, the photograph was displayed for 6 s, then SAM scales were provided for evaluating the photograph. These time intervals were selected as a result of the analysis of several scientific articles on this topic [1]. 

This experimental model showed suboptimal efficiency. The exposure time of the pictures turned out to be very short. As a result, we decided to increase the duration of the presentation of affective images and use images with extreme degrees of valence.

### 5.2. Principal Stage:

For the main research model, we selected the sets that simulate the impact of an external information image/representation:

1. 24 sound stimuli, 12 of which are extremely negative and 12 are extremely positive. The stimuli were selected based on responses according to the SAM method. Average indicators were taken from an experiment previously conducted on Russian subjects (See Appendix B)
2. 24 images, 12 of which are very negative and 12 are very positive based on SAM responses. (See Appendix B).

These affective stimuli, both auditory and visual, were randomly formed into separate blocks:

- Negative sound-with 12 negative sounds;
- Positive sound-with 12 positive sounds;
- Negative video sequence-with 12 negative images;
- Positive video sequence-with 12 positive images.

Next, we used the SAM method to evaluate the parameters of the sets. For this, we invited 30 people aged 20 to 23 (24 women and six men). Fifteen people were invited for the experiment with sound and 15 for the video sequence. Like the previous group, these individuals did not have any pronounced health problems, and they also signed informed consent to participate in the experiment. The experiment took place under similar conditions. The subjects were asked to listen/watch the set and then evaluate it using the SAM method. Thus, the valence of the resulting stimulus materials was confirmed.

For the next and principal research part, we decided to use the following research procedure. Four audiovisual stimulus materials were compiled based on the sets obtained during the previous stage.

- Negative Video (PS-)-with 12 negative sounds and images;
- Positive Video (P + S +)-with 12 positive sounds and images;
- Dissonant Video 1 (P-S +)-with 12 negative images and 12 positive sounds;
- Dissonant Video 2 (P + S-)-with 12 positive images and 12 negative sounds.

We decided to run two separate tests using the aforementioned sets:

1. Basic, with consistently lined up negative and positive videos
2. Dissonance, With dissonant videos 1 and 2 sequentially lined up for presentation.

The main stage of the study involved 37 people aged 17 to 56. Fifteen individuals were in the Dissonance set and 22 in the Basic set. Ten of the individuals were women and 15 were men. Twenty individuals gave standard SAM responses.

Like the previous subjects, the subjects did not have any pronounced health problems, and they also signed an informed consent to participate. The experiment took place under similar conditions. 

During the study, the subjects were wearing event-communication telemetry sensors. After the placing the sensor, a calibration orthopedic test was carried out to register event-related telemetry of the heart rate. The telemetry was registered while sitting and standing. Each recording lasted 3 min. Three minutes was given to track the state of the parasympathetic and sympathetic systems, because it takes 100 s to register their values, and then the equipment tracked the dynamics of these systems in segments of 10 s. The subjects were then asked to take a test to monitor the level of their emotional maladjustment (EML).

After that, a basic or dissonant complex of audiovisual sets was presented to the subjects. After each set, the subjects were asked to rate their impression of the images using the SAM method, as well as EML. Each block consisted of 12 images and 12 sounds presented in parallel. Before each presentation there was a break of 1 s. The audiovisual set was shown for 15 s; for 10 of them, the image flickered, and the sound decreased its volume to attract the subject’s attention and move on to the next set. During the breaks between images, the screen turned red for negative images and green for positive images in order to enhance the stimulus effect. Break color was based on scientific literature data. Red is an active color; green is a calming color. The research procedure lasted approximately 24 min.

## 6. Results

**Figure 7:**
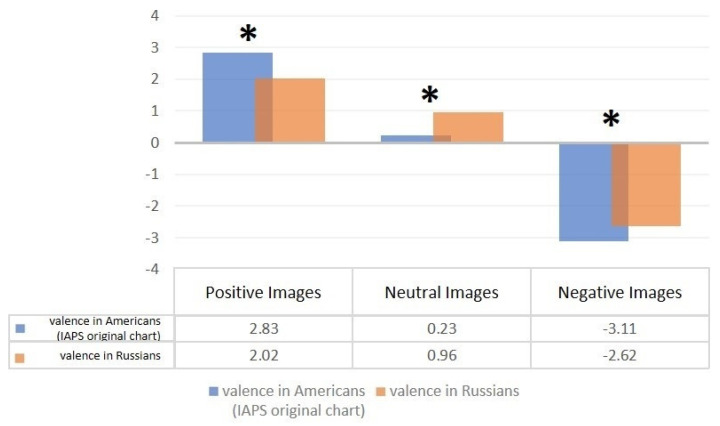
Comparison of mean values of IAPS stimulus valence between the American and Russian samples using the Wilcoxon signed–rank test.

The selected IAPS material allowed us to determine the mean values for the valence scale in the American and Russian samples (Appendix C). A special symbol (*) marks stimuli with significant differences (*p* < 0.05) according to the Wilcoxon signed-rank test (Figure 7).

We can reliably say that positive images in the Russian sample were assessed less positively, negative images less negatively, and neutral ones more positively than in the original sample.

**Figure 8:**
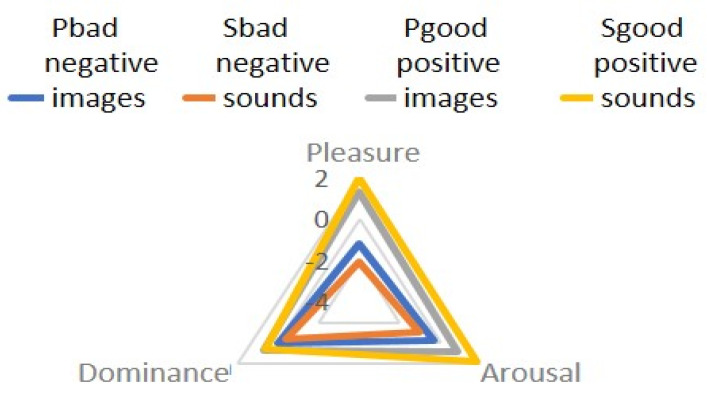
Comparison of the mean valence values of IAPS stimuli for each block-negative video (picBAD) and positive (picGood), negative audio (sndBad), and positive (sndGood).

**Figure 9:**
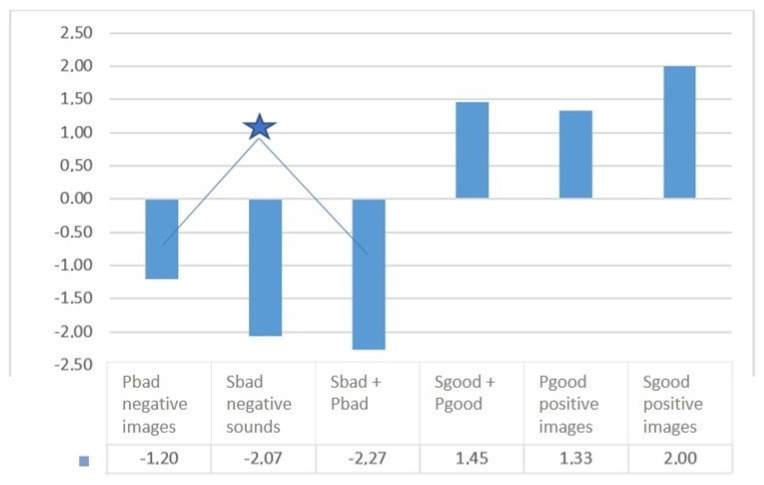
The mean values of stimuli valence for different modalities (visual–pictures; audial–sounds), comparison with the paired Wilcoxon W−test. The star indicates significant differences (*p* < 0.05).

**Figure 10:**
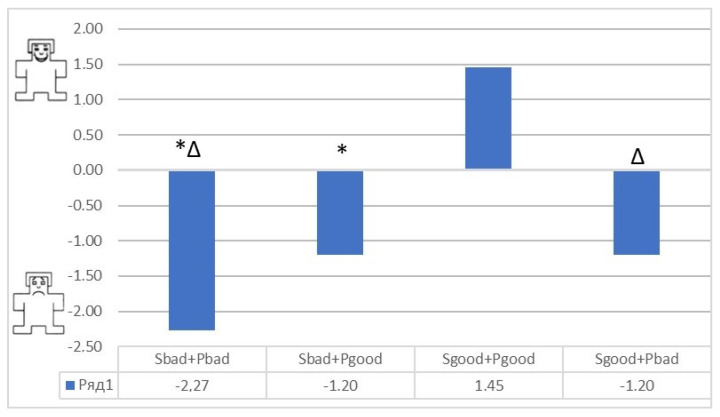
Comparison of the mean values of the stimuli valence in the Basic and Dissonance complexes. Special symbols indicate significant differences in valence according to the Wilcoxon signed−rank test: *−significant differences (*p* < 0.05) between the valence values associated with the presentation of stimuli Sbad + Pbad and Sbad + Pgood. ∆−significant differences (*p* < 0.05) between the valence values associated with the presentation of stimuli Sbad + Pbad and Sgood + Pgood.

When comparing unimodal heterovalent stimuli combined into sets of 12 images and 12 sounds, significant differences were found in the comparison between the assessment of the valence of negative and positive video sequences, as well as negative and positive audio sequences (Figure 8, Figure 9 and Figure 10).

We compared the mean values of assessments on the Valence scale for the sets with different modalities and parts of the Basic Complex (negative and positive audiovisual series)–Table 1.

The mean values and standard errors of the mean estimates of Valence for stimuli from the Base and Dissonance complexes are as follows: 
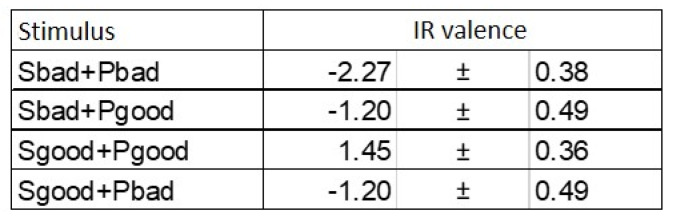


Upon presentation of the Dissonance set, the absolute value of the assessment on the “valence” scale decreased, and a reduction of emotions was observed. At the same time, the study revealed a tendency of the subjects to a negative assessment of these stimulus sets. Regardless of the type of presentation of multimodal stimuli in this complex, equal negative values were obtained in the mean values for the sample (−1.2). It turns out that the addition of any negative stimulus, whether a visual informational image or an auditory one, inclines subjects (in a subjective assessment) to a negative attitude towards the environment.

### 6.1. Influence of the Interaction of Different-Modal Affective Stimuli on the Level of Emotional Maladjustment

Let us consider the interaction of different-modal affective stimuli on the level of emotional maladjustment as one of the important elements accompanying the stressful state of an individual. 

**Figure 11:**
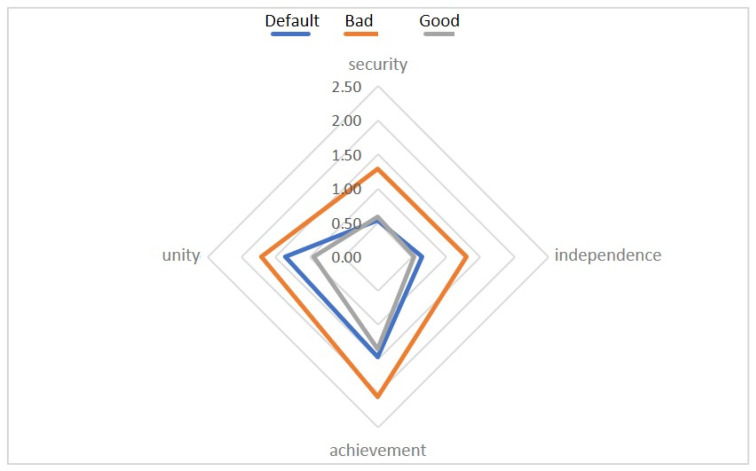
The mean values of the emotional disadaptation level (EDL Test) for the Basic complex, compared by context.

**Figure 12:**
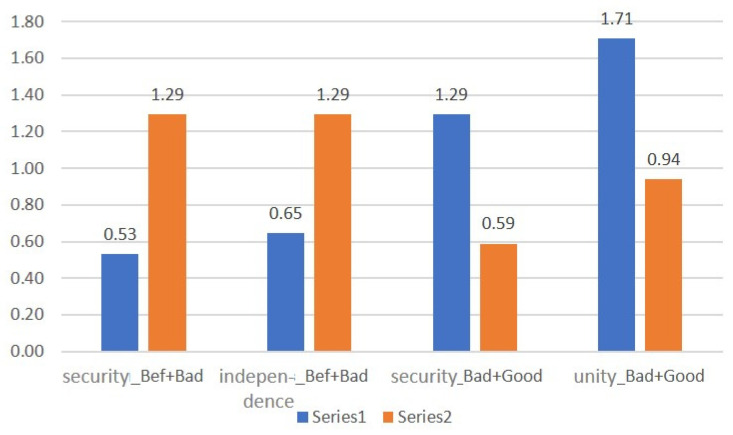
Comparison of the values of the scales with significant differences.

For the basic scenario, we have (Figure 11 and Figure 12):

When compared using the Wilcoxon W-test, significant differences were found in the Safety and Independence scales in comparison with pre-stimulation and after negative stimulation, as well as in Safety and Unity after negative and positive stimulation. It is noteworthy that in this comparison, no significant differences were found between Bad and Good sets.

The level of emotional maladjustment in the Basic Set.

- During the experiment, the maladjustment was mild;
- After negative stimulation, there was an increase in EML;
- After the positive stimulation, the levels returned to their original state;
- People with high initial maladjustment are characterized by deviant reactions to monovalent audiovisual stimuli.

This provides certain opportunities for identifying markers for diagnosing conditions in the future.

**Figure 13:**
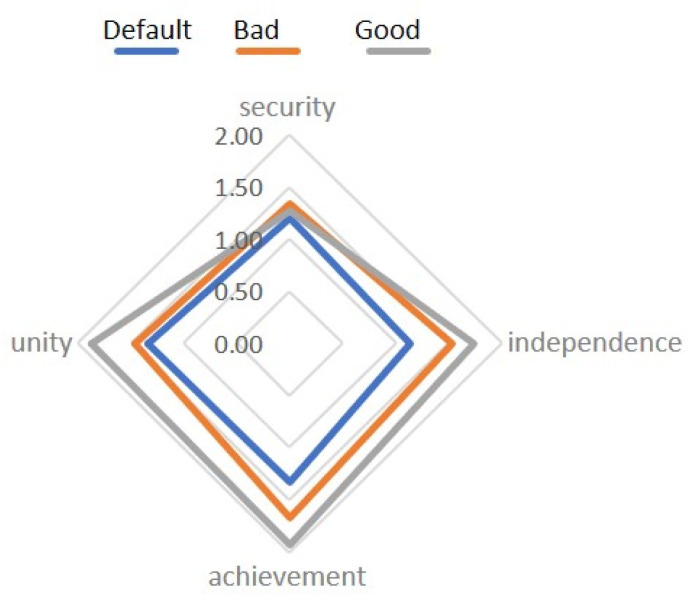
The mean values of the emotional disadaptation level (EDL Test) for the Dissonance complex, compared by context.

**Figure 14:**
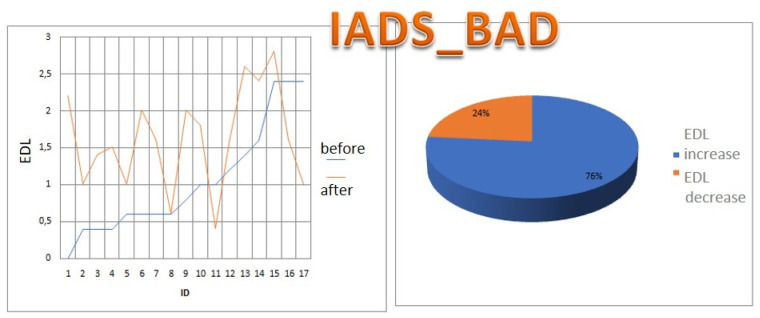
The mean values of the emotional disadaptation level (EDL Test) before and after negative stimulation.

**Figure 15:**
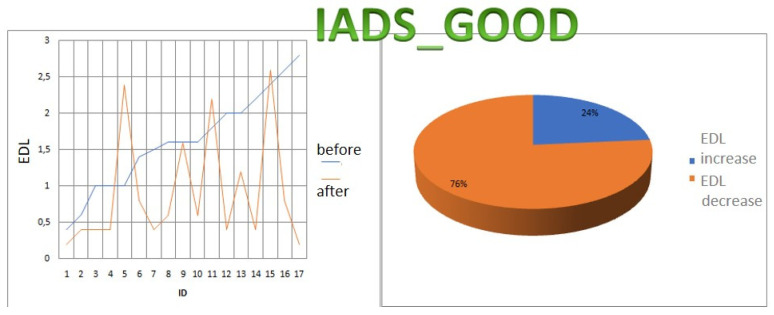
The mean values of the emotional disadaptation level (EDL Test) before and after positive stimulation.

No significant differences were found between ‘before’ and ‘after’ stimulation. During the experiment, the maladjustment was mild; 

The results of the “Dissonance” set (Figure 13):

When compared using the Wilcoxon W-test, one significant difference was found on the Unity scale before stimulation and after combining a positive image with a negative one (Figure 14 and Figure 15).

People with high initial maladjustment are characterized by deviant reactions to monovalent audiovisual stimuli.

### 6.2. Influence of the Interaction of Multi-Modal Affective Stimuli on the Functional State

Now let us consider the effect of the stimuli on the functional state of individuals. 

At the end of data collection, we performed an alignment of data. The subjects with a high proportion of artifacts were excluded from further analysis. However, the percentage of excluded test subjects did not exceed 5%, which indicates the good quality of the data obtained. 

The next step was to select subjects with standard responses according to the SAM method. We selected the subjects who rated the sets of negative images on the Valence scale from −2 to −4 and the sets of positive images from 2 to 4. The result was 30 subjects. The ration of subjects with standard answers was 67%, with non-standard-33%. Non-standard variants of perception of affective images included assessing positive and negative images as neutral, and positive images as negative. However, no one assessed negative images as positive. 

**Figure 16:**
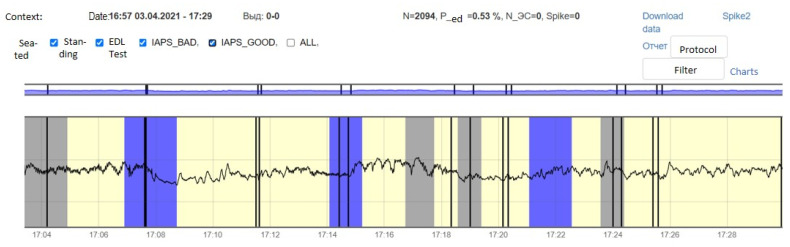
Inversion (optimization) case.

**Figure 17:**
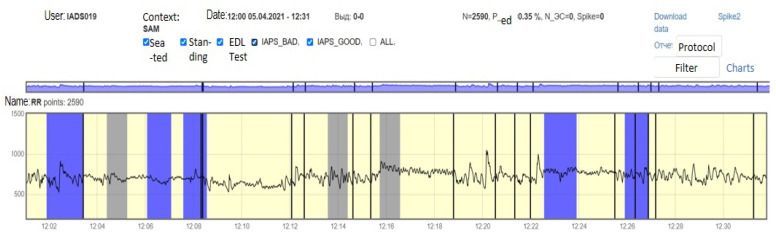
Calm state case.

**Figure 18:**
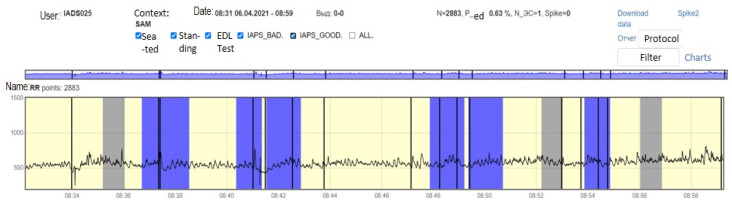
Worsening state case.

For each subject with standard responses according to SAM scales, we calculated the indicators associated with blocks of negative, positive images, and resting-state (Figure 16, Figure 17 and Figure 18):

(1) Average heart rate (HR);
(2) Average RR-intervals;
(3) Average power of the low-frequency band of the total HRV spectrum (LF);
(4) Average power of the high-frequency band of the total HRV spectrum (HF);
(5) Average ratio of the slow and fast band of the general HRV spectrum (a measure of sympathovagal balance) (LF / HF);
(6) Average power of the HRV spectrum (TP);
(7) Stress Index (SI);
(8) Functional reserve (FR);
(9) Stress Index (SN).

**Figure 19:**
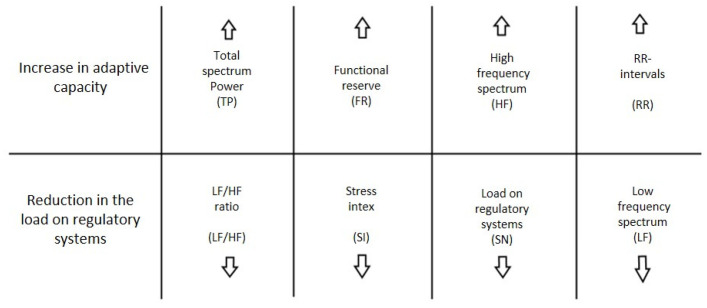
State optimization markers depending on the functional effect.

**Figure 20:**
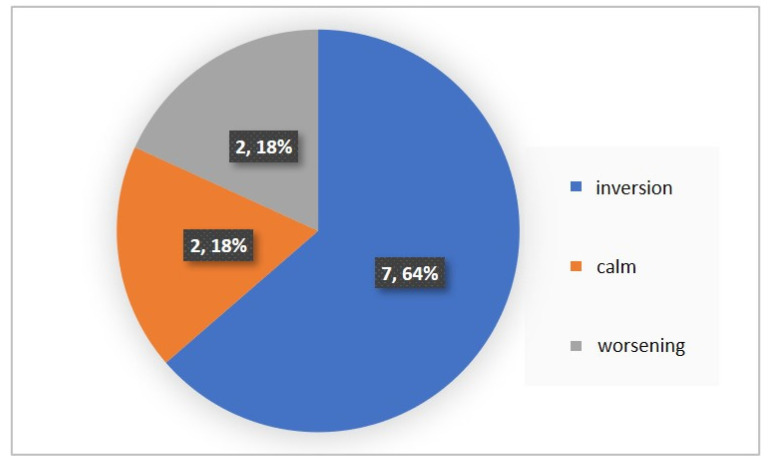
Percentage of responses to stimuli in volunteers when using the Basic complex.

**Figure 21:**
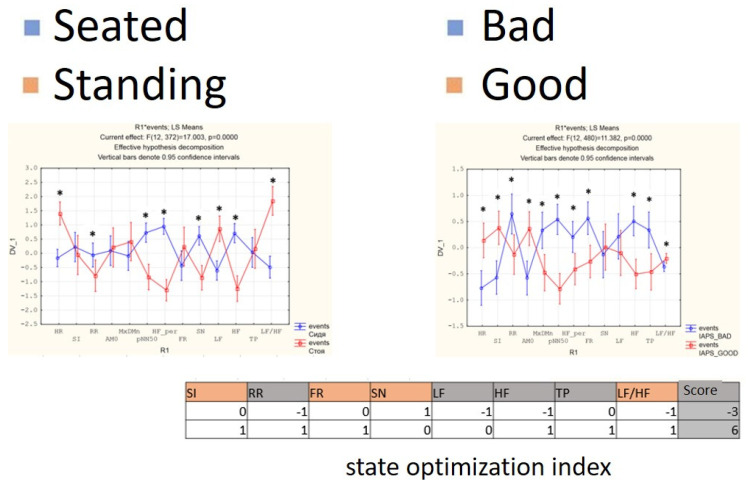
An example of inverse reaction of a test subject, the Basic complex.

**Figure 22:**
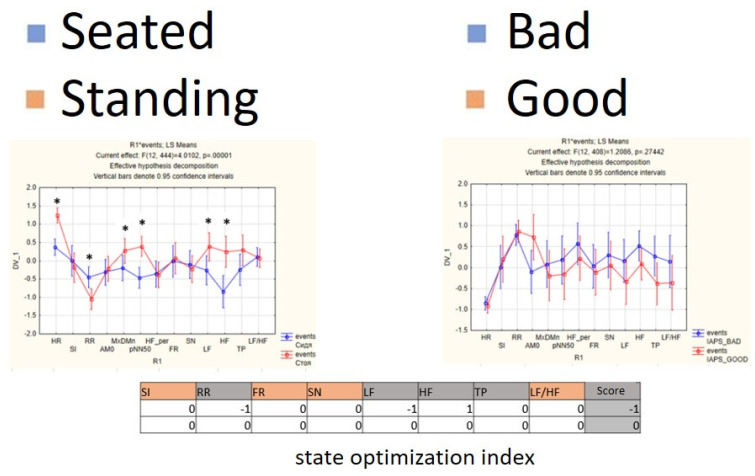
An example of a calm state of a test subject, the Basic complex.

**Figure 23:**
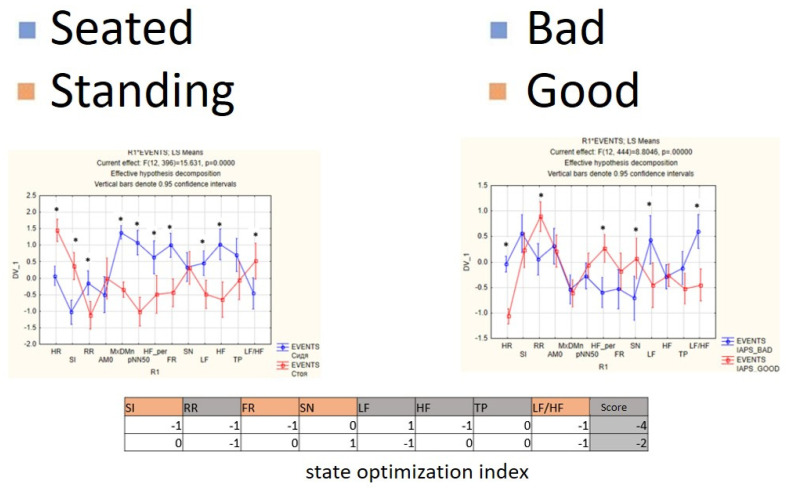
An example of a worsening state in a volunteer, the Basic complex.

**Figure 24:**
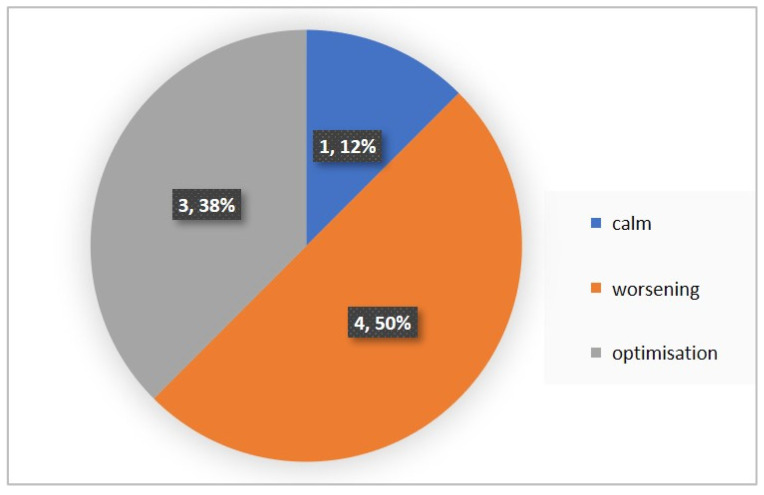
Percentage of responses to stimuli in volunteers when using the Dissonance complex.

**Figure 25:**
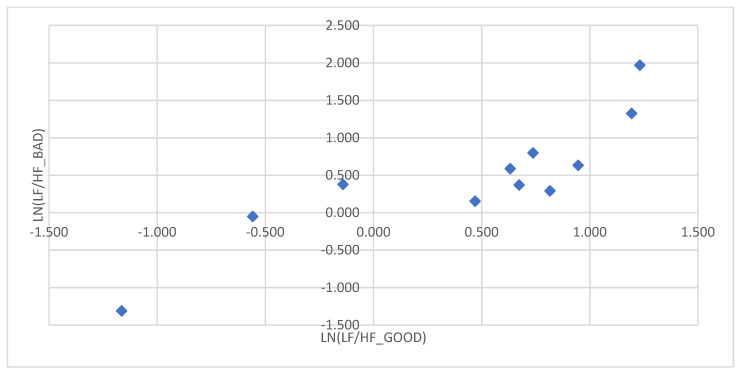
The ratios of the natural logarithms of the mean values of the vegetative balance index in different blocks in the Dissonance Set.

We attempted to simplify the spectral analysis by introducing the State Optimization Index compiled by taking the value of the spectral characteristics and indices associated with the optimization of the functional state as 1 or −1 in the presence of significant differences in the ANOVA analysis with data standardization (Figure 19, Figure 20, Figure 21, Figure 22, Figure 23, Figure 24 and Figure 25).

**Figure 26:**
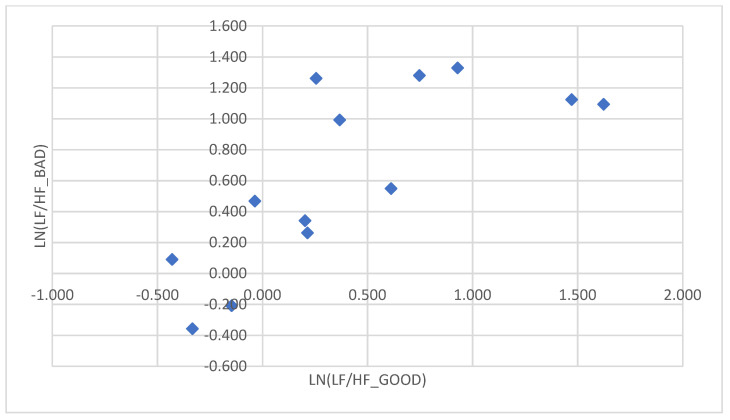
The ratios of the natural logarithms of the mean values of the vegetative balance index in different blocks in the Basic Set.

This graph shows that the sympathetic system dominates in response to the presentation of each block (Figure 26).

This graph indicates that the sympathetic system dominates in response to the presentation of both positive and negative sets.

In the majority of subjects, during the playback of both sets, the sympathetic system was activated for each of the sets. There were no significant differences according to the Student’s *t*−test between the state of the vegetative balance index during the first and second sets in both complexes.

## 7. Discussion and Conclusions

It was revealed that the addition of positive visual information images/representations (in the form of photos) significantly affects the emotional perception of audio IR. Mixing blocks in a dissonant complex produces average results, but does not affect the perception of an image. In the first case, because the image is attractive, we improve the perception of emotions associated with negative sounds, and in the second, because of the image, we spoil the impression of sounds.

Possible reasons for this effect are:

1. The module of one emotional system dampens the effect of another.

The pattern affects one emotional component, freeing it from another modality. The components interact with each other and, at some point, the modality sign becomes irrelevant. Thus, their interaction most likely becomes a consequence of the work of the limbic system. 

2. Noise (for example, the noise of cars outside the window) makes it difficult to recognize stimuli, which in turn makes it difficult to isolate an emotional image.

Thus, there exists an interaction of information (emotional) images, their interference, including for multi-modal images:

This is manifested in the reduction of emotions under the influence of asymmetric affective visual and sound images, and their invariance. The reduction of emotions was registered during the interaction of affective stimuli of different valences.

At the same time, the combination of monovalent stimuli with negative ones leads to an increase in the reaction, yet the same was not observed with positive stimuli. 

People with high initial maladjustment are characterized by deviant reactions to monovalent audiovisual stimuli. This makes it possible to diagnose the state of maladjustment based on an individual’s response to monovalent stimuli. 

When using the Dissonant audiovisual complex, a negative auditory stimulus causes an increase in the level of maladjustment. However, the video sequence does not have the same effect.

Also, the IAPS and IADS stimulus databases represent a vast corpora of affective images and sounds. These databases can be used to study various effects of affective content on the state and behavior of subjects and help in new works devoted to the topic of identifying emotional markers. However, it is necessary to note one shortcoming of these databases – the images and sounds are assessed only using the SAM method. They do not take into account the sequence in which the stimuli are presented or the way they change. Neither do they consider the operation of the visual and auditory systems, as well as the initial state of the subjects. All this makes it impossible to display an individual response to stimuli in a complex without additional means.

Taking into account the problems listed above, the authors developed a methodological complex, which includes methods for:

(1) self-diagnosis of the emotional state of the subject (SAM);
(2) the level of emotional maladjustment (EML);
(3) telemetry of the heart rate.

This helped to identify markers of affective stimuli based on the emotional image they induce, the degree of stress before and during the experiment (developed foundations of the logic of the theory of information images/representations), as well as the functional state throughout the entire duration of the experiment.

An interesting effect was found according to which Americans, on average, experience stronger emotions from different types of stimuli compared to residents of Russia. This can be attributed to a number of reasons:

(a) This could be due to the specific mental characteristics of the population and the corresponding consequences in the perception of various information stimuli.
(b) Also, the conditions of the experiment cannot be ideally reproduced, and the difference in the result can be explained by a slightly different (possibly more constraining) situation.
(c) This could also be due to the peculiarities of the sample, its insufficient representativeness.

In addition, we proposed a model of neurocognitive activity of the brain based on the theory of information images/representations. This model is instrumental for interpreting ambiguous emotions and could be developed further to simulate and predict some special cases. At this stage, however, it serves as a tool to represent the human mind and the foundation of an experimental paradigm that assists with the interpretation of the results.

## Figures and Tables

**Table 1:** Significant variations in stimuli of different modalities (visual–photos; audial–sounds) and different valence (positive, negative); comparison with the paired Wilcoxon W-test.

Comparison Pairs	Valid	T	Z	*p*-Value
Sbad+Pbad & Sbad+Pgood	11	3.00	2.67	0.01
Sbad+Pbad & Sgood + Pbad	21	9.00	3.70	0.00
Sbad+Pbad & Sgood + Pgood	13	9.00	2.55	0.01
Sbad+Pbad & Pbad	11	0.00	2.93	0.00
Sbad+Pbad & Sbad	12	10.50	2.24	0.03
Sbad+Pbad & Pgood	15	0.00	3.41	0.00
Sbad+Pbad & Sgood	15	0.00	3.41	0.00
Sbad+Pgood & Sgood + Pbad	15	4.00	3.18	0.00
Sbad+Pgood & Pgood	13	2.00	3.04	0.00
Sbad+Pgood & Sgood	14	1.50	3.20	0.00
Sgood+Pbad & Sgood + Pgood	14	4.50	3.01	0.00
Sgood+Pbad & Pbad	15	2.00	3.29	0.00
Sgood+Pbad & Sbad	15	1.00	3.35	0.00
Sgood+Pgood & Pgood	13	7.50	2.66	0.01
Sgood+Pgood & Sgood	13	0.00	3.18	0.00
Pbad & Pgood	14	2.00	3.17	0.00
Pbad & Sgood	15	3.00	3.24	0.00
Sbad & Pgood	15	0.00	3.41	0.00
Sbad & Sgood	14	0.00	3.30	0.00

## Data Availability

Web-platform COGNITOM (cogni-nn.ru).

## List of Abbreviations and Conventions

Heart rate (HR)	heart rate
RR	RR-intervals
LF	the power of the spectrum at low frequencies
HF	power of the spectrum in the high-frequency region
LF/HF	ratio of spectrum powers in the region of low and high frequencies (vegetative balance index)
TP	the power of the HRV spectrum
SI	stress index
FR	functional reserve
SN	voltage degree (Amo/2Mo×mxDMn)
AM0	the amplitude of the mode
MxDMn	variation range
pNN50	the number in the percentage of pairs of the last normal intervals (without extrasystoles), differing by more than 50 MC
HF_per	the percentage of the spectrum in the high-frequency region
IAPS	International Affective Picture System (international system of affective pictures)
IADS	International Affective Digitized Sound system
SAM	Self-Assessment Manikin (model for self-assessment of emotions)
LEM	Monitoring the level of emotional maladjustment
IR/II	informational representations/images

## Appendix A

**Table A1:** Responses of subjects according to the SAM questionnaire to stimulus material from the IAPS database based on an American sample.

IAPS, №	Name	Group	Valence	Arousal
2209	Bride	POS	2.64	0.59
1710	Puppies	POS	3.34	0.41
2045	Baby	POS	2.87	0.47
2070	Baby	POS	3.17	−0.49
2216	Children	POS	2.57	0.83
2340	Family	POS	3.03	−0.1
2347	Children	POS	2.83	0.56
2550	Couple	POS	2.77	−0.32
4220	EroticFemale	POS	3.02	2.17
4626	Wedding	POS	2.6	0.78
5480	Fireworks	POS	2.53	0.48
5825	Sea	POS	3.03	0.46
5830	Sunset	POS	3	−0.08
5833	Beach	POS	3.22	0.71
5910	Fireworks	POS	2.8	0.59
7330	IceCream	POS	2.69	0.14
7502	Castle	POS	2.75	0.91
8170	Sailboat	POS	2.63	1.12
8190	Skier	POS	3.1	1.28
8200	WaterSkier	POS	2.54	1.35
8210	Boat	POS	2.53	0.94
8370	Rafting	POS	2.77	1.73
8420	Tubing	POS	2.76	0.56
8470	Gymnast	POS	2.74	1.14
8496	WaterSlide	POS	2.58	0.79
8499	Rollercoaster	POS	2.63	1.07
8501	Money	POS	2.91	1.44
8502	Money	POS	2.51	0.78
2095	Toddler	NEG	−3.21	0.25
2205	Hospital	NEG	−3.05	−0.47
2345.1	BlackEye	NEG	−2.74	0.5
2352.2	BloodyKiss	NEG	−2.91	1.25
2375.1	Woman	NEG	−2.8	−0.12
3000	Mutilation	NEG	−3.41	2.34
3001	HeadlessBody	NEG	−3.38	1.64
3005.1	OpenGrave	NEG	−3.37	1.2
3010	Mutilation	NEG	−3.29	2.16
3100	BurnVictim	NEG	−3.4	1.49
3103	Injury	NEG	−2.93	1.06
3140	DeadBody	NEG	−3.17	1.36
3195	Stitches	NEG	−2.94	1.36
3261	Tumor	NEG	−3.18	0.75
3266	Injury	NEG	−3.44	1.79
3301	InjuredChild	NEG	−3.2	0.21
3350	Infant	NEG	−3.12	0.72
3530	Attack	NEG	−3.2	1.82
9040	StarvingChild	NEG	−3.33	0.82
9140	Cow	NEG	−2.81	0.38
9183	HurtDog	NEG	−3.31	1.58
9187	InjuredDog	NEG	−3.19	1.45
9300	Dirty	NEG	−2.74	1
9325	Vomit	NEG	−3.11	1.01
9405	SlicedHand	NEG	−3.17	1.08
9570	Dog	NEG	−3.32	1.14
9571	Cat	NEG	−3.04	0.64
9635.1	ManOnFire	NEG	−3.1	1.54
1026	Snake	NEUT	−0.91	0.61
1390	Bees	NEUT	−0.5	0.29
1505	DogRace	NEUT	−0.87	−0.27
1560	Hawk	NEUT	0.97	0.51
1645	Wolf	NEUT	−0.01	0.14
1820	Crocodile	NEUT	0.35	0.67
1908	Jellyfish	NEUT	0.28	−0.12
1931	Shark	NEUT	−1	1.8
2018	VeiledWoman	NEUT	0.56	−0.08
2122	TongueOut	NEUT	0.15	−0.41
2220	MaleFace	NEUT	0.03	−0.07
2606	Dance	NEUT	0.92	−0.22
4071	AttractiveFem	NEUT	0.97	0.14
4460	EroticMale	NEUT	0.6	−0.06
5455	Cockpit	NEUT	0.79	−0.44
5661	Cave	NEUT	0.96	−0.85
5920	Volcano	NEUT	0.16	1.23
5940	Lava	NEUT	−0.77	1.29
5950	Lightning	NEUT	0.99	1.79
6000	Prison	NEUT	−0.96	−0.09
6910	Bomber	NEUT	0.31	0.62
7240	Gym	NEUT	1.02	0.51
7402	Pastry	NEUT	0.98	0.05
7476	Ramen	NEUT	−0.01	−0.37
7497	Crowd	NEUT	0.19	−0.03
7560	Freeway	NEUT	−0.53	0.24
7600	Dragon	NEUT	0.9	0.5
7640	Skyscraper	NEUT	0	1.03

## Appendix B

**Table A2:** Responses of subjects according to the SAM questionnaire to stimulus material from the IAPS database based on a Russian sample.

IAPS, №	Name	Group	Valence (Rus)	Arousal (Rus)	Dominance (Rus)
3266	Injury	NEG	−3.44	−0.88	−0.23
3000	Mutilation	NEG	−3.52	0.02	−0.02
3100	BurnVictim	NEG	−3.38	−0.16	0.63
3001	HeadlessBody	NEG	−2.55	0.09	0.08
3005.1	OpenGrave	NEG	−3.53	−0.23	−0.01
9040	StarvingChild	NEG	−2.26	0.14	0.67
9570	Dog	NEG	−3.41	−0.9	−0.05
9183	HurtDog	NEG	−2.91	0.19	0.04
2095	Toddler	NEG	−1.54	1.73	1.46
3010	Mutilation	NEG	−3.38	−0.7	0.86
3530	Attack	NEG	−1.88	−0.28	1.58
3301	InjuredChild	NEG	−2.11	0.47	1.22
9187	InjuredDog	NEG	−2.89	−1	0.23
3261	Tumor	NEG	−3.48	0.56	0.02
9405	SlicedHand	NEG	−2.26	0.4	0.69
3140	DeadBody	NEG	−3.5	0.27	0.54
3350	Infant	NEG	−1.13	−0.64	0.7
9325	Vomit	NEG	−1.68	−0.71	0.76
9635.1	ManOnFire	NEG	−2.19	−0.02	1.51
2205	Hospital	NEG	−2.77	−0.21	0.93
9571	Cat	NEG	−3.44	−0.41	0.67
3195	Stitches	NEG	−2.81	1.19	0.9
3103	Injury	NEG	−2.89	1.19	1.3
2352.2	BloodyKiss	NEG	−2.16	−1.49	0.53
9140	Cow	NEG	−3.24	0.18	0.58
2375.1	Woman	NEG	−0.76	1.16	2.24
9300	Dirty	NEG	−1.55	−0.42	1.27
2345.1	BlackEye	NEG	−2.84	1.61	1.27
7476	Ramen	NEUT	2.96	−0.4	1.68
7402	Pastry	NEUT	2.79	0.74	2.25
4071	AttractiveFem	NEUT	2.56	−0.02	2.15
5661	Cave	NEUT	2.55	0.49	2.89
7600	Dragon	NEUT	2.26	0.52	2.9
1560	Hawk	NEUT	1.89	0.53	2.87
1390	Bees	NEUT	1.83	0.34	2.45
1908	Jellyfish	NEUT	1.75	−0.27	2.32
7560	Freeway	NEUT	1.71	−0.24	2.61
5950	Lightning	NEUT	1.65	0.35	2.41
2018	VeiledWoman	NEUT	1.53	0.16	2.74
5455	Cockpit	NEUT	1.26	0.84	3.07
1026	Snake	NEUT	1.23	0.55	2.28
1505	DogRace	NEUT	1.18	0.79	2.39
1820	Crocodile	NEUT	1.05	0.03	2.52
2606	Dance	NEUT	1.03	0.86	2.83
1645	Wolf	NEUT	0.79	−0.07	2.23
7497	Crowd	NEUT	0.61	0.21	2.71
7240	Gym	NEUT	0.58	1.02	2.98
1931	Shark	NEUT	0.57	0.15	2.32
6000	Prison	NEUT	0.49	0.1	2.86
2122	TongueOut	NEUT	0.33	0.66	2.88
5920	Volcano	NEUT	0.29	1.1	2.49
4460	EroticMale	NEUT	−0.42	1.13	2.77
6910	Bomber	NEUT	−0.46	−0.14	2.61
2220	MaleFace	NEUT	−0.56	0.18	2.77
7640	Skyscraper	NEUT	−1.32	2.22	1.52
5940	Lava	NEUT	−1.51	1.61	2.29
1710	Puppies	POS	3.46	0.11	1.61
7330	IceCream	POS	3.26	0.13	1.79
4220	EroticFemale	POS	3.1	0.59	2.21
5833	Beach	POS	3.1	0.06	1.75
5825	Sea	POS	2.58	1.84	2.12
8170	Sailboat	POS	2.41	1.2	2.41
2550	Couple	POS	2.36	2	2.32
8190	Skier	POS	2.31	0.37	2.85
7502	Castle	POS	2.25	1.64	2.62
2347	Children	POS	2.23	1.67	2.09
2045	Baby	POS	2.21	0.59	2.76
2340	Family	POS	2.2	0.16	1.93
2209	Bride	POS	2.16	1.38	2.55
8370	Rafting	POS	2.12	0.97	2.63
5830	Sunset	POS	2.09	0.54	1.57
8210	Boat	POS	2.08	1.18	2.29
2070	Baby	POS	1.86	0.78	1.97
5910	Fireworks	POS	1.85	0.85	2.72
4626	Wedding	POS	1.84	0.34	2.72
8420	Tubing	POS	1.5	0.06	2.34
5480	Fireworks	POS	1.43	1.05	3.05
8496	WaterSlide	POS	1.41	0.27	2.17
8470	Gymnast	POS	1.33	0.14	2.48
8499	Rollercoaster	POS	1.21	0.5	2.45
8501	Money	POS	1.21	0.42	2.26
2216	Children	POS	1.16	0.95	2.14
8502	Money	POS	0.88	−0.06	1.97
8200	WaterSkier	POS	0.86	1.73	2.65

## Appendix C

**Table A3:** Selection of incentive material according to IASP, taking into account the Russian sample.

IAPS, №	Name	Group	Valmn (Eng)	Aromn (Eng)	Arosd (Eng)	Valence (Rus)	Arousal (Rus)	Dominance (Rus)
1710	Puppies	POS	3.34	0.41	−2.66	3.46	0.11	1.61
7330	IceCream	POS	2.69	0.14	−2.42	3.26	0.13	1.79
4220	Erotic Female	POS	3.02	2.17	−2.31	3.1	0.59	2.21
5833	Beach	POS	3.22	0.71	−2.34	3.1	0.06	1.75
8170	Sailboat	POS	2.63	1.12	−2.7	2.41	1.2	2.41
2550	Couple	POS	2.77	−0.32	−2.57	2.36	2	2.32
8190	Skier	POS	3.1	1.28	−2.43	2.31	0.37	2.85
7502	Castle	POS	2.75	0.91	−2.69	2.25	1.64	2.62
2347	Children	POS	2.83	0.56	−2.66	2.23	1.67	2.09
2340	Family	POS	3.03	−0.1	−2.8	2.2	0.16	1.93
2209	Bride	POS	2.64	0.59	−2.63	2.16	1.38	2.55
8370	Rafting	POS	2.77	1.73	−2.76	2.12	0.97	2.63
3005.1	Open Grave	NEG	−3.37	1.2	−2.46	−3.53	−0.23	−0.01
3000	Mutilation	NEG	−3.41	2.34	−2.73	−3.52	0.02	−0.02
3140	DeadBody	NEG	−3.17	1.36	−3.03	−3.5	0.27	0.54
3261	Tumor	NEG	−3.18	0.75	−2.36	−3.48	0.56	0.02
3266	Injury	NEG	−3.44	1.79	−2.91	−3.44	−0.88	−0.23
9571	Cat	NEG	−3.04	0.64	−2.5	−3.44	−0.41	0.67
9570	Dog	NEG	−3.32	1.14	−2.69	−3.41	−0.9	−0.05
3010	Mutilation	NEG	−3.21	2.26	−3.14	−3.38	−0.7	0.86
3100	Burn Victim	NEG	−3.4	1.49	−2.77	−3.38	−0.16	0.63
9140	Cow	NEG	−2.81	0.38	−2.81	−3.24	0.18	0.58
9183	HurtDog	NEG	−3.31	1.58	−2.88	−2.91	0.19	0.04
3103	Injury	NEG	−2.93	1.06	−2.7	−2.89	1.19	1.3
